# Hybrid Nanocomposites of Tenoxicam: Layered Double Hydroxides (LDHs) vs. Hydroxyapatite (HAP) Inorganic Carriers

**DOI:** 10.3390/molecules28104035

**Published:** 2023-05-11

**Authors:** Lauretta Maggi, Valeria Friuli, Giovanna Bruni, Alessia Rinaldi, Marcella Bini

**Affiliations:** 1 Department of Drug Sciences, University of Pavia, Viale Taramelli 12, 27100 Pavia, Italy; lauretta.maggi@unipv.it; 2 Department of Chemistry, University of Pavia, Viale Taramelli 16, 27100 Pavia, Italy; giovanna.bruni@unipv.it (G.B.); marcella.bini@unipv.it (M.B.); 3 CSGI, Department of Chemistry, University of Pavia, Viale Taramelli 16, 27100 Pavia, Italy; 4 Nanocarbon Laboratory, Department of Mathematical, Physics and Informatics Sciences, University of Parma, Parco Area delle Scienze 7/A, 43124 Parma, Italy; alessia.rinaldi@unipr.it; 5 National Reference Centre for Electrochemical Energy Storage (GISEL)—INSTM, Via G. Giusti 9, 50121 Firenze, Italy

**Keywords:** tenoxicam, drug delivery, in vitro dissolution studies, LDHs, HAP

## Abstract

The search for effective systems to facilitate the release of poorly bioavailable drugs is a forefront topic for the pharmaceutical market. Materials constituted by inorganic matrices and drugs represent one of the latest research strategies in the development of new drug alternatives. Our aim was to obtain hybrid nanocomposites of Tenoxicam, an insoluble nonsteroidal anti-inflammatory drug, with both layered double hydroxides (LDHs) and hydroxyapatite (HAP). The physicochemical characterization on the base of X-ray powder diffraction, SEM/EDS, DSC and FT-IR measurements was useful to verify the possible hybrids formation. In both cases, the hybrids formed, but it seemed that the drug intercalation in LDH was low and, in fact, the hybrid was not effective in improving the pharmacokinetic properties of the drug alone. On the contrary, the HAP–Tenoxicam hybrid, compared to the drug alone and to a simple physical mixture, showed an excellent improvement in wettability and solubility and a very significant increase in the release rate in all the tested biorelevant fluids. It delivers the entire daily dose of 20 mg in about 10 min.

## 1. Introduction

The formulation of poorly water-soluble drugs is challenging for pharmaceutical scientists and an issue which is due to rise, since at least 40% of the newly developed drugs are, indeed, not very soluble in water [1]. When a drug is orally administered in solid dosage forms, such as tablets, capsules, or suspension, it must be released and dissolved in the gastrointestinal fluids before its absorption. The bioavailability of many poorly water-soluble drugs is limited by their dissolution rate, which is controlled by the useful surface area: when the dissolution rate of the drug is slower than the absorption rate, the dissolution becomes the rate-limiting step [2]. Therefore, numerous attempts have been made to improve the dissolution of certain drugs to obtain a more rapid and complete absorption. To this aim, salt formation, complexation, micelles, liposomes, particle size reduction, and solid dispersion were some of the contrived methods [2,3,4]. Lately, one of the most promising systems for the pharmaceutical industry that is attracting considerable attention is represented by inorganic–organic hybrids formed by a host inorganic matrix and a drug guest [5,6]. In this regard, hydroxyapatites and layered double hydroxides are certainly very interesting inorganic matrices. Hydroxyapatite (*HAP*), Ca_10_(PO_4_)_6_(OH)_2_, is the main constituent of biological tissues, such as bones and teeth [7]. It has several intriguing features, such as biocompatibility, biodegradability, osteogenesis, osteoconductivity and bioactivity, and can form direct bonds with living tissues. HAP has many different applications, for example in tissue engineering, nanomedicine, industrial catalysis and in orthopedic implant coating [8,9]. In this last case, it was demonstrated that the use of Ca-deficient hydroxyapatite could favour osseointegration [9]. *HAP* nano-spheres, thanks to their low solubility in physiological conditions, can be used as carriers for controlled and localized drug delivery. In fact, *HAP* easily binds to both positive and negative molecules by simple absorption [10,11,12,13]. The toxicity to other organs is minimized by using hydroxyapatite for drug delivery; the drug concentration in the blood is reduced and a repeated administration of drugs is less necessary. Layered double hydroxides (*LDHs*) are a well-known class of anionic clays, named hydrotalcites, with chemical formula [M^2+^_1−x_ M^3+^_x_ (OH)_2_](A^n−^)_x/n_ yH_2_O, with M^2+^ (Mg, Zn, Ni, Co or Cu), M^3+^(Al, Cr, Sc, Ga, Gd or Fe) and A^n−^ (CO_3_^2−^, NO_3_^−^, Cl^−^), these latter anions to balance positive charges of the hydroxide layers. *LDHs*, as *HAP*, are biocompatible, biodegradable and minimally toxic. Hydrotalcite is yet present in commercial antacid formulation (e.g., Talcid^®^), thanks to its basic behavior. The LDHs properties are various and strictly dependent on the precursors used for the synthesis (organic or inorganic), so it is possible to apply them in many different fields, from catalysis, adsorption of organic wastes or metal ions to electrochemistry and nanomedicine [14,15,16]. The interest of these materials lies in the easiness of substitution of the cited anions with different species, so obtaining plenty of hybrids, which can improve both the dissolution rate and/or solubility of the drugs in different media [17,18,19,20].

The non-steroidal anti-inflammatory drugs (NSAID) pertaining to the oxicam class are especially poorly soluble. One of them, Tenoxicam (*Tnx*), possesses analgesic and antipyretic properties and can be used for osteoarthritis and rheumatoid arthrosis as well as for brief treatments of muscoloskeletal pains [21]. Four crystalline polymorphic forms are reported in the literature, together with an amorphous form and an acetonitrile solvate [22,23,24]. Form III is commercialized; it melts at about 217 °C and then decomposes. Tenoxicam belongs to the class II of the Biopharmaceutical Classification System [25]: it is well absorbed but practically insoluble in water, creating hindrances in its in vivo oral bioavailability and development of injection formulations [26]. It is slightly soluble in other solvents, such as ethyl acetate, ethane diol, 2-(2-ethoxyethoxy)ethanol, ethanol, 1-butanol and 2-butanol [27]. Some approaches have been evaluated to improve the solubility and dissolution of *Tnx*, such as fast dissolving tablets, cosolvency and solid dispersions [28,29,30]. In this regard, for this challenging active principle, the hybrids formation could represent an advantageous approach.

Our aim was to synthesize, hybrids of Tenoxicam with both *LDH* and *HAP* inorganic hosts. To our knowledge, this is the first attempt to develop this type of hybrids. A complete physical-chemical characterization was performed to prove the hybrids formation by means of X-ray powder diffraction (XRPD), Fourier transform infrared spectrometer (FT-IR), differential scanning calorimetry (DSC) and scanning electron microscopy with energy dispersive spectroscopy (SEM-EDS). Then, pharmaceutical measurements, in particular of the dissolution rates in different media simulating the gastrointestinal environments, and of solubility and contact angle, allowed us to test the suitability of the hybrids as drug delivery systems.

## 2. Results

The hybrids were characterized by the combined use of different physical-chemical techniques, to first verify their formation and then the possible improvement of the dissolution rate and solubility with respect to the drug alone. In the following section, we will discuss the XRPD, FT-IR, DSC and SEM-EDS data and, separately, the pharmaceutical results.

### 2.1. Physical-Chemical Characterization of LDH-Tenoxicam

**Figure 1:**
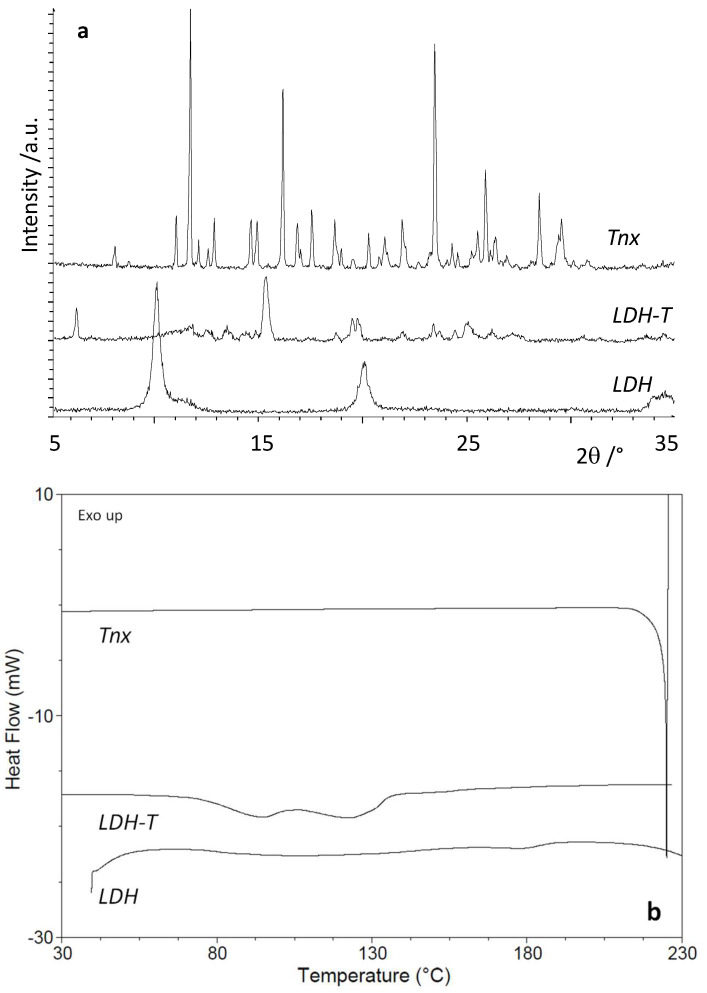

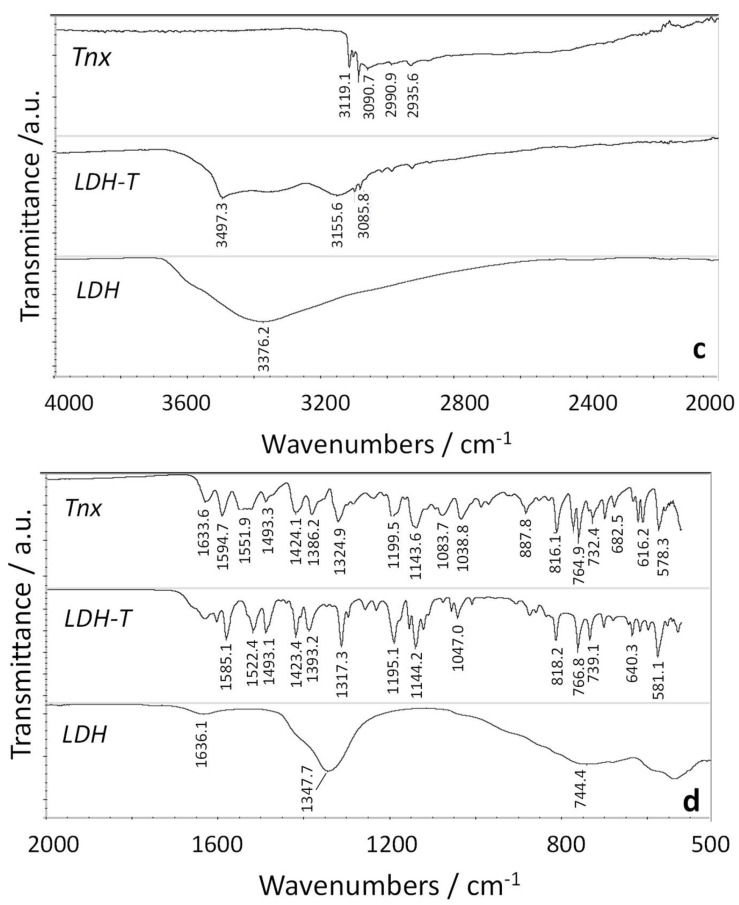
(**a**) XRPD patterns, (**b**) DSC curves and (**c**,**d**) FT-IR spectra of *Tnx*, *LDH* and *LDH-T* samples.

XRPD is one of the most suitable techniques to verify the drug intercalation in the *LDH* structure. The shifts to lower angles of the *00l* reflections, which is a characteristic of *LDHs*, and due to the anions exchange (typically nitrates/carbonates with drug anions), should be analysed to identify the possible intercalation. In Figure 1a, the XRPD patterns of *Tnx, LDH* and *LDH-T* are compared. *Tnx* is a well crystalline drug, as demonstrated by the narrow diffraction peaks; this is the polymorphic Form III as proved by the main peaks at about 11.7°, 16.2° and 23.5° [22,23], without traces of other forms.

The *LDH* pattern shows the reflections expected for a layered material, which agree with those of the reference hydrotalcite pattern [31]. The most intense peaks are those of the basal diffraction of *00l* reflections of *LDHs*, with the NO_3_^−^ as intercalated anions, due to the employed reagents. The small peak near to the main reflection at about 10° can suggest the intercalation of some CO_3_^2−^, notwithstanding the use of decarbonated solvents and N_2_ flux during the synthesis. From the peak position of the *003* reflection (2θ = 10.1°) it is possible to calculate an interplanar distance of 8.75 Å, in agreement with literature data [17,31].

The pattern of the *LDH-T* hybrid shows a lot of peaks, two of them corresponding to the *003* and *006* reflections (6.2° and 12.5°), shifted to lower angle with respect to those of the pure *LDH*, due to drug intercalation. The other peaks are difficult to assign with confidence. They do not resemble those of *Tnx*, so we can hypothesize that the formation of an intermediate phase deriving from the interaction of *LDH* and *Tnx,* however different from the hybrid expected if the intercalation was the only involved phenomenon. Therefore, the *LDH-T* pattern could suggest that the intercalation was not effective, as will be confirmed by the dissolution study results (see later).

Thermal data, in particular the melting or decomposition temperatures of drugs, are another useful proof of the formation of new chemical entities as a consequence of drug intercalation. In Figure 1b, the DSC curves of *Tnx*, *LDH* and *LDH-T* samples are shown. *Tnx* is stable up to its melting point, as demonstrated by the endothermic event at about 211 °C, followed by a strongly exothermic decomposition, in agreement with the literature data of Form III [22]. *LDH* does not show relevant thermal events: the small and broad endothermic peaks between 130 °C and 180 °C can suggest an initial de-hydroxylation of the hydroxides layers and water release [17]. However, these phenomena are very limited, suggesting a high stability of the hydrotalcite host in the entire analyzed temperature range, as expected. In the *LDH-T* curve, no thermal events attributed to *Tnx* are visible. On the contrary, two endothermic peaks (at about 100 °C and 150 °C) are present, which could be due to the release of residual solvents or water molecules as well as to an initial de-hydroxylation, as for *LDH,* or to a new crystalline phase. This last hypothesis would confirm the XRPD results.

**Scheme 1:**
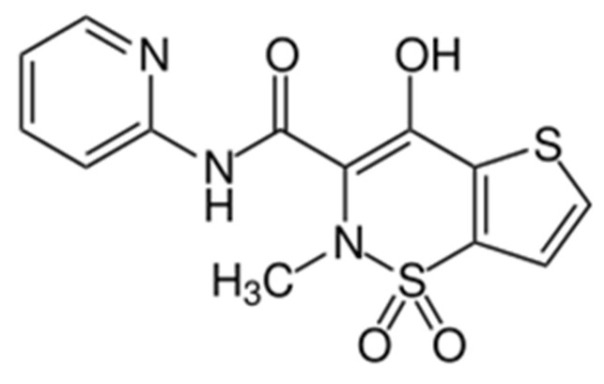
Molecular structure of Tenoxicam.

The FT-IR spectra of *Tnx*, *LDH* and *LDH-T* are reported in Figure 1c (4000–2000 cm^−1^) and Figure 1d (2000–500 cm^−1^). The *Tnx* spectrum shows the main absorption at 3200–3000 cm^−1^ (OH stretching, NH stretching and =CH stretching), 1633 cm^−1^ (C=O stretching: amide I band), 1594 cm^−1^ (aromatic ring), 1552 cm^−1^ (NH bending: amide II band), 1324 and 1195 cm^−1^ (SO_2_ antisymmetric and symmetric stretching) (see Scheme 1 showing the molecular structure of the drug).

For what concerns the pure *LDH*, the broad band at about 3376 cm^−1^ is due to the stretching of hydroxyl groups, the peak at 1348 cm^−1^ could be attributed to nitrate groups, while the peaks under 800 cm^−1^ are assigned to metal–oxygen and metal hydroxide stretching modes [17].

In the *LDH-T* spectrum, the band at 1348 cm^−1^ of pure *LDH* is absent and some differences with respect to the spectrum of *Tnx* can be noticed. For example, the peaks of the amide and of the SO_2_ groups are shifted suggesting that in the *LDH-T* sample an interaction between the drug and *LDH* took place (see also XRPD and DSC data).

**Figure 2:**
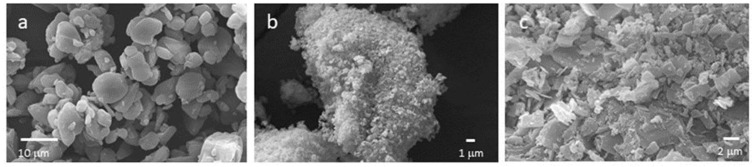
SEM images of (**a**) *Tnx*, (**b**) *LDH* and (**c**) *LDH-T* samples.

The morphological analysis was performed by means of SEM microscopy (Figure 2). *Tnx* (Figure 2a) is constituted by particles of irregular shapes with variable dimensions, up to about 10 μm. *LDH* (Figure 2b) is formed by aggregates of small particles, with a flat shape, as expected for layered compounds [31]. The *LDH-T* morphology (Figure 2c) is different from both the drug and *LDH*. Aggregates of large particles, with a flat shape, resembling those of *LDH* but with clear edges, are present. The observed morphologies are similar to those of comparable hybrids and *LDHs* [32].

### 2.2. Physical-Chemical Characterization of HAP-Tenoxicam

**Figure 3:**
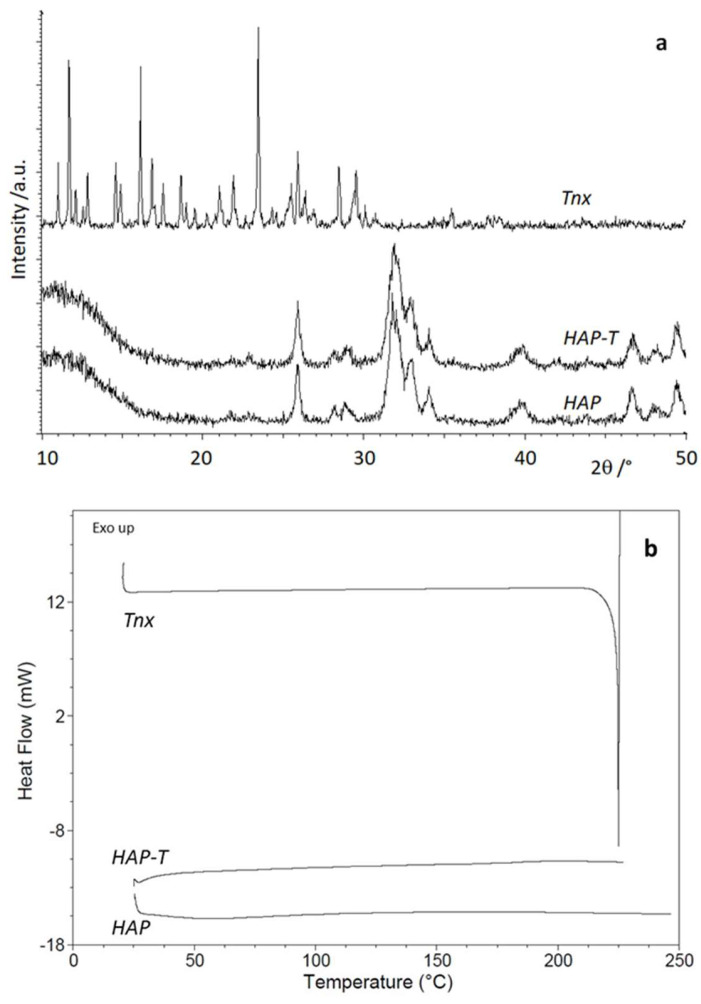
(**a**) XRPD patterns and (**b**) DSC curves of *Tnx*, *HAP* and *HAP-T* samples.

The patterns of *Tnx*, *HAP* and *HAP-T* are shown in Figure 3a.

The pattern of *Tnx* has been already described (see Section 2.1). The peak positions of *HAP* well agree with those expected for Ca_10_(PO_4_)_6_(OH)_2_ phase (Card N. 74-0565), with a hexagonal lattice and the *P6_3_/m* space group [13]. The XRPD pattern of *HAP-T* is over imposable to that of *HAP*: all the peaks pertain to the *HAP* phase, and no peaks of Tenoxicam can be seen. This can be due to the presence of the drug as amorphous and/or in very low amount.

The DSC analysis was performed on *Tnx* (whose DSC curve was also reported in Figure 1b and described in Section 2.1), *HAP* and *HAP-T* (Figure 3b). *HAP* is a stable material, which, in the analysed temperature range, only shows a broad endothermic peak at low temperature due to the release of adsorbed water [12]. *HAP-T* also does not present thermal events: the melting peak of the drug is not visible, so supporting the hypothesis that, if the drug is present in the hybrid, it has to be amorphous (see XRPD results).

**Figure 4:**
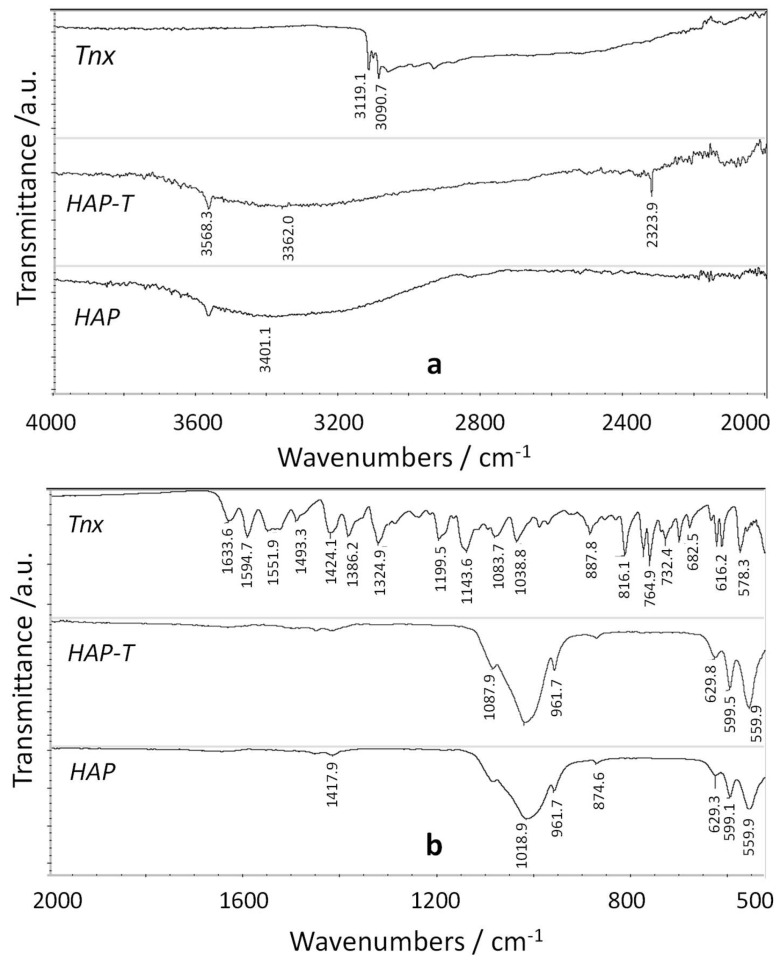
FT-IR spectra of *Tnx*, *HAP* and *HAP-T* in the spectral ranges (**a**) 4000–2000 cm^−1^ and (**b**) 2000–500 cm^−1^.

The FT-IR spectra for *Tnx*, *HAP* and *HAP-T* samples are reported in Figure 4a,b.

The FT-IR spectrum of the drug was yet described in Section 2.1. The *HAP* spectrum shows some typical vibrational bands: those at 560 cm^−1^ and 600 cm^−1^ are due to the bending of O-P-O group, the band at 962 cm^−1^ to the stretching of PO_4_^3−^ group and the bands at around 1000 cm^−1^ to P-O antisymmetric stretching. The peaks at 3400 cm^−1^ and 630 cm^−1^ were attributed to O–H stretching and bending, respectively. Additionally, the occurrence of bands at around 1400 cm^−1^ suggests that *HAP* is carbonated [33]. The inclusion of CO_3_^2–^ ions was due to the CO_2_ present in the environment during the synthesis. It has been reported that carbonated *HAP* offered better bioactivity [33].

The *HAP-T* spectrum is practically over imposable to that of pure *HAP* (Figure 4). No peaks clearly attributable to the drug can be evidenced, suggesting that the adsorbed drug could be present in small amount, lower than the detection limit of the analysis.

**Figure 5:**
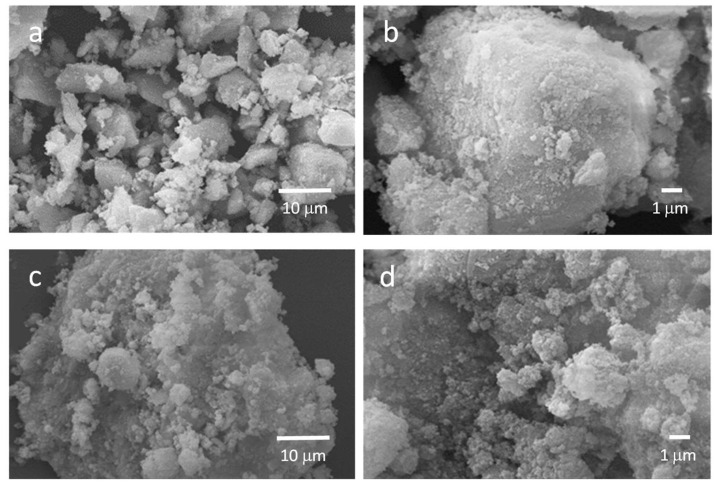
SEM images at two different magnifications of *HAP* (**a**,**b**) and *HAP-T* (**c**,**d**) samples.

The SEM images of *HAP* and *HAP-T* are reported in Figure 5a,b and Figure 5c,d respectively (the micrograph of *Tnx* is shown in Figure 2a).

Pure *HAP* (Figure 5a,b) appears to be constituted by large particles of irregular shapes. At higher magnification it is possible to observe small, rounded particles, well aggregated. *HAP-T* hybrid (Figure 5c,d) has an analogous morphology. It is composed of larger particles than those of *HAP*, that at high magnification are likewise aggregates of small, rounded particles. Drug particles which are typical (Figure 2a) cannot be identified.

**Figure 6:**
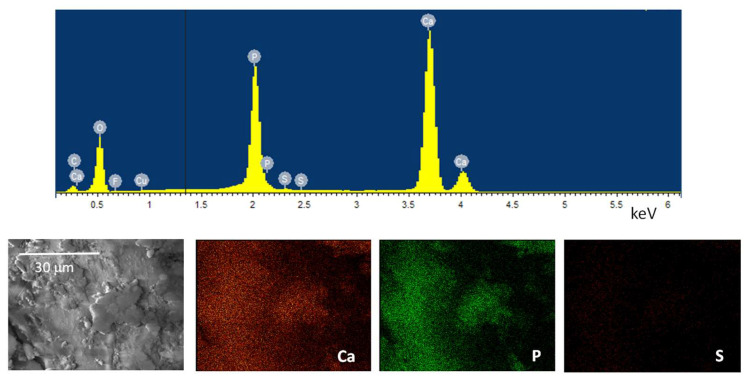
EDS spectrum, SEM micrograph and corresponding maps of Ca, P and S of *HAP-T* sample.

The EDS microanalysis, combined with SEM can ascertain the hybrids formation by analyzing the presence of the characteristic elements of *HAP* (Ca and P ions) and Tenoxicam (S ions). The EDS spectrum and the corresponding SEM image, together with the maps of the relevant elements of the *HAP-T* sample are reported in Figure 6. From the spectrum, we can identify the expected elements (with traces of impurities) and a homogeneous distribution of Ca and P from the corresponding maps. The S ions are barely visible due to their low amount (Figure 6), but they are distributed homogeneously in the sample, so supporting the hypothesis that a host–guest interaction took place.

The elements amount can be obtained from the EDS spectrum (Table 1), so to calculate the chemical composition of calcium phosphate, which was found to be in agreement with the expected hydroxyapatite stoichiometry (Ca/P ratio = 1.67). In addition, we can also estimate the amount of the absorbed drug, which is about 4.7 wt%. To find this value, we used the molecular weight (MW) of hydroxyapatite, 1004 g/mol, which however could be underestimated considering that FT-IR revealed some amount of carbonate ions. Therefore, the actual drug percentage might be lower than that calculated (see Section 2.3.1).

### 2.3. Pharmaceutical Results

#### 2.3.1. Drug Loading

The drug loading in the two hybrids was 16.0 ± 4.7% *w*/*w* for *LDH-T* and 2.2 ± 0.2% *w*/*w*, for *HAP-T,* so lower for this latter.

#### 2.3.2. Dissolution Tests

**Figure 7:**
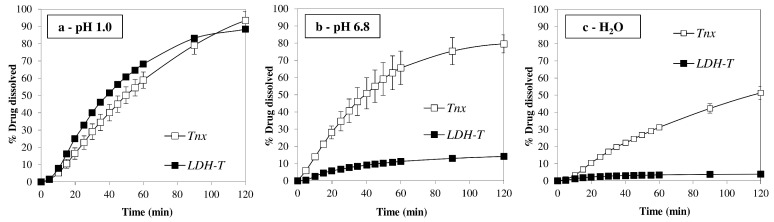
Dissolution profiles of *Tnx* and *LDH-T* in pH 1.0 (**a**), pH 6.8 buffer (**b**) and in deionised water (**c**). All samples contain a dose of 20 mg of Tenoxicam.

The dissolution profiles of *LDH-T* showed no improvement in the dissolution rate of *Tnx*. It is known that the drug is more soluble at pH 1.0. At this pH, the behavior of *LDH-T* followed that of the pure active principle, while in the other two fluids the release rate of Tenoxicam is even lower or much lower than the reference (Figure 7).

**Figure 8:**
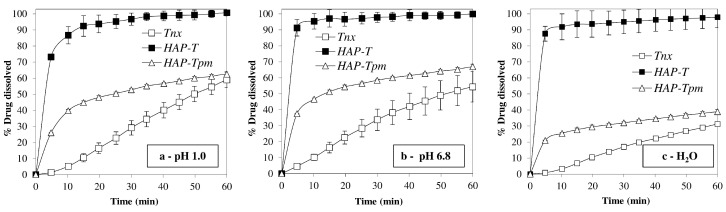
Dissolution profiles of *Tnx*, *HAP-T* and the physical mixture *HAP-Tpm* in pH 1.0 (**a**), pH 6.8 buffer (**b**) and deionised water (**c**). All samples contain a dose of 20 mg of Tenoxicam.

On the contrary, *HAP-T* showed a remarkable increase in the dissolution rate of *Tnx* in all the fluids, with no direct, apparent relation with the pH value or the presence of a buffer (Figure 8). This fact is particularly important in oral administration, where a drug encounters extremely different environmental conditions affecting these two variables.

This result appears even more comforting when compared with the physical mixture of the two components: although the presence of the *HAP* in the mixture could partially promote the wettability of the *Tnx*, it was not able to reach the dissolution rate of the *HAP-T* hybrid.

#### 2.3.3. Solubility and Contact Angle

The equilibrium solubility of *Tnx* reached after 24 h was 43.3 ± 5.1 mg/L. The equilibrium solubility of *HAP-T* was instead >170 mg/L (the test was stopped when the suspension become too thick, like sludge), higher than more than eight times the dose.

**Figure 9:**
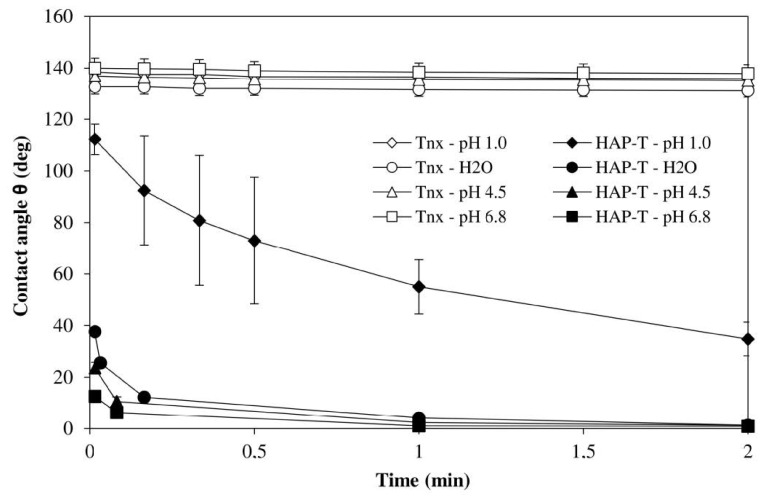
Contact angle values (θ), as a function of time, for *Tnx* and *HAP-T* in the different media considered (pH 1.0, H_2_O, pH 4.5 and pH 6.8).

The contact angle measurements confirmed that one of the factors determining the increase in the dissolution rate of *HAP-T* was the considerable improvement in its wettability compared to the drug alone (Figure 9).

In less than ten seconds, *HAP-T* was completely wetted by three out of the four considered fluids, but also at pH 1.0, although with a slower rate, the contact angle approached zero in about two minutes. On the contrary, *Tnx* showed a very high contact angle which did not change significantly with time. Additionally, in this case, the pH of the medium does not seem to influence significantly the phenomenon.

## 3. Discussion

The synthesis of inorganic-organic hybrids represents a new strategy of drug delivery, very appealing for the pharmaceutical market, particularly for poorly water-soluble drugs. Our work was devoted to the preparation of two hybrids of Tenoxicam, a NSAID poorly soluble in water, with two different inorganic hosts: a layered double hydroxide and hydroxyapatite. The physical-chemical characterization of the hybrids allowed us to verify their formation, but the real proof of the effectiveness of hybrids in improving the drug biopharmaceutical properties was the dissolution test under conditions that can simulate the passage through the gastro-intestinal tract. The *LDH-T* hybrid, from UV-Vis spectroscopy, seemed to load a satisfactory amount of drug, which in principle could be positive for the realization of efficient pharmaceuticals forms. Unfortunately, the dissolution rates in the tested media were lower with respect to the Tenoxicam alone, apart from in HCl, pH 1.0, in which the profiles were comparable: however, at this pH, the drug is more soluble. We could hypothesize, on the basis of the characterization that was carried out, that the drug intercalation was very limited, as demonstrated by XRPD data. In fact, a mixture of phases formed: the hybrid compound, in small amounts, together with an intermediate phase with a different XRPD pattern and different thermal behavior with respect to *Tnx* and *LDH*. Therefore, the dissolution rate of the hybrid showed no enhancement due to the failure of the intercalation and the formation of a phase with similar dissolution characteristics to the drug itself. The issues in obtaining the intercalated phase could be due to the low solubility of Tenoxicam, which is scarcely dissolved and is not maintained in solution for long time.

The results obtained from the other hybrid, *HAP-T,* are instead, very positive. Despite the loading being lower with respect to *LDH-T*, the dissolution rate was much increased compared to both the drug alone and the physical mixture of the two components. Water solubility also increased more than eight times the dose, although it was not possible to complete the evaluation for practical reasons (the suspension tended to become too thick). Another determining factor in improving the dissolution performance of *HAP-T* was certainly its wettability. The contact angle rapidly decreased in a few seconds or minutes confirming a considerably better performance than *Tnx*, which, showed instead, a very high contact angle that did not decrease as a function of time. The hybrid contained low amount of the drug in the amorphous form, as revealed by both XRPD and DSC data, homogeneously distributed in the sample. Our observations suggest that this hybrid could represent a promising system for the enhanced delivery of Tenoxicam from oral dosage forms.

## 4. Materials and Methods

### 4.1. Syntheses

Tenoxicam (*Tnx*) was gently donated by Olon (Casaletto Lodigiano, LO, Italy).

The drug was very poorly soluble in water [27], so it was necessary for the hybrids’ preparation to find a suitable medium for its solubilisation. After many trials, the best-identified medium was a mixture of 3:1 *v*/*v* ethanol/water.

#### 4.1.1. LDH-Tenoxicam

To obtain the hybrid, it was necessary to first identify the proper *LDH* for the intercalation. Many trials were performed by changing the divalent cations (Mg^2+^ or Zn^2+^), their ratio (3:1 or 2:1), and the kind of synthetic route (co-precipitation, reconstruction, ionic exchange). Finally, the chosen *LDH* for the intercalation was the Zn_3_Al-LDH obtained by a modified co-precipitation method. The proper amount of Zn(NO_3_)_2_ 6H_2_O and Al(NO_3_)_3_ 6H_2_O were weighted and placed in a three necks balloons under N_2_ flux, then 30 mL of anhydrous ethanol and 10 mL of decarbonated water (the ratio required for the *Tnx* solubilization) were added. The solution was stirred and when the reagents were completely solubilized, NaOH 2M was added to reach a pH of about 8. The Tenoxicam was then added under stirring in an amount equal to 1.5 times the mol amount of Zn, keeping the pH at 8 (adjusting with NaOH if needed). The obtained dispersion was stirred in N_2_ flux for 24 h at room temperature, then it was centrifuged at 6000 rpm for 5 min and the recovered powder was washed with decarbonated water and dried in oven at 50 °C overnight. This sample will be named *LDH-T*.

A pure Zn_3_Al-LDH sample was obtained by co-precipitation route in the same way, but using 30 mL of water as solvent, maintaining all the other conditions. This sample will be named *LDH*.

#### 4.1.2. HAP-Tenoxicam

The synthesis of the hybrid at first required the preparation of hydroxyapatite Ca_10_(PO_4_)_6_ (OH)_2_: the co-precipitation method, which is rapid and easily scalable, was chosen [34]. Stoichiometric amounts of Ca(NO_3_)_2_ 4H_2_O and (NH_4_)_2_HPO_4_ were dissolved separately in 30 mL of water with magnetic stirring. Both solutions were basified with NH_4_OH until a pH of about 10–11 was reached. After about 15 min, the solution of phosphate was slowly added drop by drop under stirring to that of calcium. The pH was again controlled and other NH_4_OH was possibly added. Then, the temperature was raised to 80 °C and maintained for 1 h. After cooling, the solution was centrifuged at 6000 rpm for 5 min, and the collected powder was washed three times with distilled water and placed in oven for 22 h at 100 °C. This sample will be named *HAP*.

For the hybrid preparation, the solution of drug was first obtained. A total of 100 mg of Tenoxicam was added to 10 mL of a solution of ethanol/water (3:1 *v*/*v*) and magnetically stirred until the drug was completely dissolved. Then 150 mg of *HAP* was added to the drug solution, that was sonicated for about 5 min to improve the *HAP* dispersion and maintained under stirring at room temperature for 24 h. Finally, the dispersion was centrifuged at 6000 rpm for 5 min and the collected powder was dried in oven at 50 °C overnight. The hybrid will be named *HAP-T*.

### 4.2. Physical-Chemical Characterizations

X-ray powder diffraction (XRPD) measurements were performed by using a Bruker D5005 diffractometer (Bruker BioSpin, Fällanden, Switzerland) with the Cu Kα radiation, graphite monochromator and scintillation detector. The patterns were collected in air with a step size of 0.03° and counting time of 2 s per step in proper angular ranges depending on the involved samples: 5–35° (*Tnx, LDH and LDH-T*) and 10–50° (*HAP, HAP-T*) by using a low background silicon sample holder.

Fourier-transformed infrared (FT-IR) spectra were obtained with a Nicolet FT-IR iS20 spectrometer (Nicolet, Madison, WI, USA) equipped with ATR (attenuated total reflectance) sampling accessory (Smart iTR with diamond plate) by co-adding 32 scans in the 4000–500 cm^−1^ range at 4 cm^−1^ resolution.

Differential-scanning calorimetry (DSC) measurements were carried out by a DSC Q2000 apparatus interfaced with a TA 5000 data station (TA Instruments, New Castle, DE, USA). The instrument was calibrated using ultrapure (99.999%) indium (m.p. = 156.6 °C; Δ*H* = 28.54 J g^−1^) as standard. The calorimetric measurements were conducted up to 250 °C at a heating rate of 5 K min^−1^ on samples amount of about 3–5 mg in open standard aluminum pans under nitrogen flow (45 mL·min^−1^).

Scanning electron microscopy (SEM) images were collected by a Zeiss Evo MA10 (Carl Zeiss, Oberkochen, Germany) microscope coupled with the energy dispersive spectroscopy (EDS) detector for microanalysis (X-max 50 mm, Oxford Instruments, Abingdon, UK). The samples for SEM analysis were sputtered with a thin layer of gold and analyzed at an acceleration voltage of the electron beam of 20 kV. The EDS data were obtained on not sputtered samples.

### 4.3. Pharmaceutical Measurements

#### 4.3.1. Drug Loading

The drug content was measured by UV-Vis absorbance (Lambda 25, Perkin-Elmer, Monza, Italy). A calibration curve was previously performed in the conditions in which the active principle is most soluble, (i.e., 0.1 N HCl, pH 1.0) at 370 nm, obtaining a correlation coefficient of 0.9999. In the same condition, the drug content of the *LDH-T* and *HAP-T* hybrids was determined. The UV spectrum of inorganic carriers was previously recorded: they do not show any absorbance at the same wavelength of *Tnx*.

#### 4.3.2. Dissolution Test

The USP apparatus 2, paddle (Erweka DT-D6, Erweka GmbH, Dusseldorf, Germany) was used to evaluate the drug dissolution profiles from the hybrid samples at 37.0 ± 0.5 °C, 50 rpm (three replicates).

All the samples, containing 20 mg of *Tnx*, previously sieved through a 230-mesh grid (63 μm), were tested in 900 mL of three the different fluids: hydrochloric solution at pH 1.0, (simulating the gastric environment in fasted conditions) pH 6.8 phosphate buffer (simulating the intestinal environment) and deionised water. All the dissolution media were prepared according to the reagent and buffer solutions section of the USP [35].

The drug concentrations were measured by a UV-Vis spectrophotometer (Lambda 25, Perkin-Elmer, Monza, Italy) on filtered portions of the dissolution fluid at 370 nm for the *Tnx* determination. The data were processed by a suitable software (Winlab V6 software, Perkin-Elmer, Monza, Italy). Since the dissolution performances of sample *LDH-T* were not satisfactory, we proceeded with the further characterization of *HAP-T* sample only. For comparison purpose, a physical mixture of the two components *Tnx* and *HAP* (called *HAP-Tpm*) was prepared in the same weight ratio obtained from the drug loading test and subjected to the dissolution tests described above.

#### 4.3.3. Solubility

The solubility of *Tnx* and *HAP-T* were determined in deionised water at 21 °C, using the shake-flask method left under magnetic stirring at 200 rpm. At predetermined time intervals, an aliquot of the supernatant of the saturated solutions was filtered (0.45 μm, Millipore) and the drug concentration was determined by spectrophotometric detection. The results were the average of three determinations. The test was repeated until equilibrium was reached.

#### 4.3.4. Contact Angle

Contact Angle Meter DMe-211Plus (NTG Nuova Tecnogalenica, Cernusco, Italy) was used to compare the wettability of *Tnx* and *HAP-T.* A 9 µL drop of different fluids (a hydrochloric 0.1 N solution at pH = 1.0, a pH 4.5 and pH 6.8 phosphate buffers and deionised water) was dropped from the needle onto the surface of the samples. The images of the drop in contact with the samples were acquired at progressive times (from t = 0 up to 300 s) and a suitable software, provided by the equipment, measured the contact angle. Three replicates were performed for each sample.

## 5. Conclusions

Tenoxicam, belonging to the class II of the Biopharmaceutical Classification System, is practically insoluble in water and many other fluids, so the search for ways to increase its bioavailability is a forefront topic for the pharmaceutical market. We demonstrated the excellent performances of the Hydroxyapatite–Tenoxicam hybrid as drug delivery system, with improved dissolutions rates and solubility of the drug in different media thanks to the enhanced wettability. As a future perspective, an increased drug loading could enable to manufacture an amazing drug-delivery system, easily synthesized, biocompatible and green.

## Figures and Tables

**Table 1:** Atomic percentages and Ca/P ratio as determined from EDS analysis.

	Ca	P	S	Ca/P Ratio
*HAP-T*	15.7	10.4	0.15	1.51

## Data Availability

Data available on request.
